# Metabolic Reprogramming in Modulating T Cell Reactive Oxygen Species Generation and Antioxidant Capacity

**DOI:** 10.3389/fimmu.2018.01075

**Published:** 2018-05-16

**Authors:** Josephin N. Rashida Gnanaprakasam, Ruohan Wu, Ruoning Wang

**Affiliations:** Center for Childhood Cancer & Blood Diseases, Hematology/Oncology & BMT, The Research Institute at Nationwide Children’s Hospital, Ohio State University, Columbus, OH, United States

**Keywords:** reactive oxygen species, oxidative stress, metabolism, T cell, antioxidant

## Abstract

A robust adaptive immune response requires a phase of proliferative burst which is followed by the polarization of T cells into relevant functional subsets. Both processes are associated with dramatically increased bioenergetics demands, biosynthetic demands, and redox demands. T cells meet these demands by rewiring their central metabolic pathways that generate energy and biosynthetic precursors by catabolizing and oxidizing nutrients into carbon dioxide. Simultaneously, oxidative metabolism also produces reactive oxygen species (ROS), which are tightly controlled by antioxidants and plays important role in regulating T cell functions. In this review, we discuss how metabolic rewiring during T cell activation influence ROS production and antioxidant capacity.

## Introduction

T cells are central orchestrators of antigen-specific adaptive immunity and tolerance. Upon stimulation of antigen receptors, T cells rapidly transit from naïve to an active state followed by massive clonal expansion. Depending on the nature of pathogens and the surrounding cytokine milieu, proliferating T cells can differentiate into diverse phenotypic and functional subsets to elicit a robust immune response. After the clearance of pathogens, the majority of effector T cells die through apoptosis and the remaining memory T (T_mem_) cells are responsible for immunity upon re-exposure to the same pathogen. Accumulating evidence suggests that a coordinated rewiring of cellular metabolism is required for T cell activation and differentiation by fulfilling the bioenergetic, biosynthetic, and redox demands (1–9). Importantly, different phenotypic and functional T cell subsets are characterized by distinct metabolic programs (Table 1), which are largely controlled by immune modulatory signaling cascades (10–17). Naïve T (T_nai_) cells, T_mem_ cells, and immune-suppressive regulatory T (T_reg_) cells predominantly rely on fatty acid oxidation (FAO) and oxidative phosphorylation (OXPHOS) to meet their relatively low-energy needs (14, 15, 18, 19). However, persistent aerobic glycolysis, the pentose phosphate pathway (PPP), and glutaminolysis are required to drive cell growth, clonal expansion, and effector functions in both CD4^+^ subsets and CD8^+^ effector T (T_eff_) cells (Table 1) (10, 15, 16, 18, 20–31).

**Table 1:** The metabolic profiles of T cell subsets.

T cell type	Naïve	Active	Differentiated	Memory T cell (T_mem_)
Metabolic profile	FAO OXPHOS	Aerobic glycolysis PPP Glutaminolysis	Th1: aerobic glycolysis/some OXPHOS Th2: aerobic glycolysis Th9: aerobic glycolysis Th17: aerobic glycolysis, glutaminolysis Tfh: aerobic glycolysis, OXPHOS T_reg_: FAO, OXPHOS CTL: aerobic glycolysis CAT: oxidation, phosphorylation	FAO OXPHOS

*FAO, fatty acid oxidation; OXPHOS, oxidative phosphorylation; PPP, pentose phosphate pathway; Th1, T helper 1 cell; Th2, T helper 2 cell; Th9, T helper 9 cell; Th17, T helper 17 cell; Tfh, follicular helper T cell; T_reg_, regulatory T cell; CTL, cytotoxic T lymphocyte; CAT, chronically activated T cell; T_mem_, memory T*.

These metabolic programs actively support ATP production by providing mitochondrial OXPHOS substrates, support biomass accumulation by generating metabolic precursors for the biosynthesis of protein, lipids, and nucleic acids, and maintain redox balance through generation and elimination of reactive oxygen species (ROS).

## Mitochondrial OXPHOS and NADPH Oxidases (NOXs) in Generating ROS in T Cells

The mitochondria are the central metabolic hub and powerhouse of all eukaryotic cells. The oxidation of acetyl-CoA to carbon dioxide (CO_2_) by the tricarboxylic acid (TCA) cycle is the central metabolic process for fueling ATP production. While glycolysis and FAO primarily provide the OXPHOX substrate, acetyl-CoA, for mitochondria in T_nai_ cells, T_mem_ cells, and T_reg_ cells (14, 15, 18, 19), heightened mitochondrial biogenesis during T cell activation leads to higher numbers of mitochondria and likely the enhanced mitochondrial dependent metabolic flux in T_eff_ cells compared with T_nai_ cells (23, 32, 33). In particular, a surplus of 3-, 4-, and 5-carbon metabolites (anaplerotic substrates) including pyruvate, malate, and α-ketoglutarate (α-KG) feed into the TCA cycle during the catabolism of glutamine and other amino acids (5, 13, 15, 34). The electron transport chain (ETC) constantly transfers electrons from NADH and FADH2 to oxygen while allowing protons (H^+^) to pass through the inner mitochondrial membrane to form an electrochemical proton gradient that drives ATP synthesis. However, both protons and electrons can leak from the ETC due to the uncoupling of ATP synthase from the proton gradient and a premature exit of electron before reaching cytochrome *c* oxidase, respectively. Electron leak largely occurs at the sites of complex I (NADH–ubiquinone oxidoreductase) and complex III (ubiquinone–cytochrome *c* oxidoreductase) in the ETC and results in the partial reduction of oxygen, generating superoxide $\left({\text{O}}_{2}^{-•}\right)$. Subsequently, mitochondrial dismutase acts to convert superoxide to hydrogen peroxide (H_2_O_2_), which is free to diffuse into cytosol and act as a redox signaling molecule to elicit different cellular responses (35–37). Thus, increased ROS production in T cells can occur as a result of metabolic reprogramming during T cell activation. Besides mitochondria, cytoplasmic ROS is generated by NOXs, which is also an important source of ROS in T cell. NOX family proteins are transmembrane proteins that transport the electrons from nicotinamide adenine dinucleotide (phosphate), NAD(P)H, to oxygen and generate superoxide anion as the intermediate product of oxidase and subsequently H_2_O_2_, as the product of dismutation of the superoxide. There are different isoforms of the NOX enzyme including NOX1, NOX2, NOX3, NOX4, NOX5, dual oxidase 1, and DUOX2, and the expression of these subunits varies among different tissues. NOX-2 is an important source of ROS in T cells (38, 39). The ROS production by NOX is regulated at various levels including the assembly of functional NOX complex, the availability of prosthetic group, flavin adenine dinucleotide, the intracellular concentration of calcium, cell surface receptor signals mediated by G protein-coupled receptors, complement, T cell receptor (TCR), and CD28 (35–37, 40, 41).

## ROS Signaling in Regulating T Cell Activation and Differentiation

**Figure 1:**
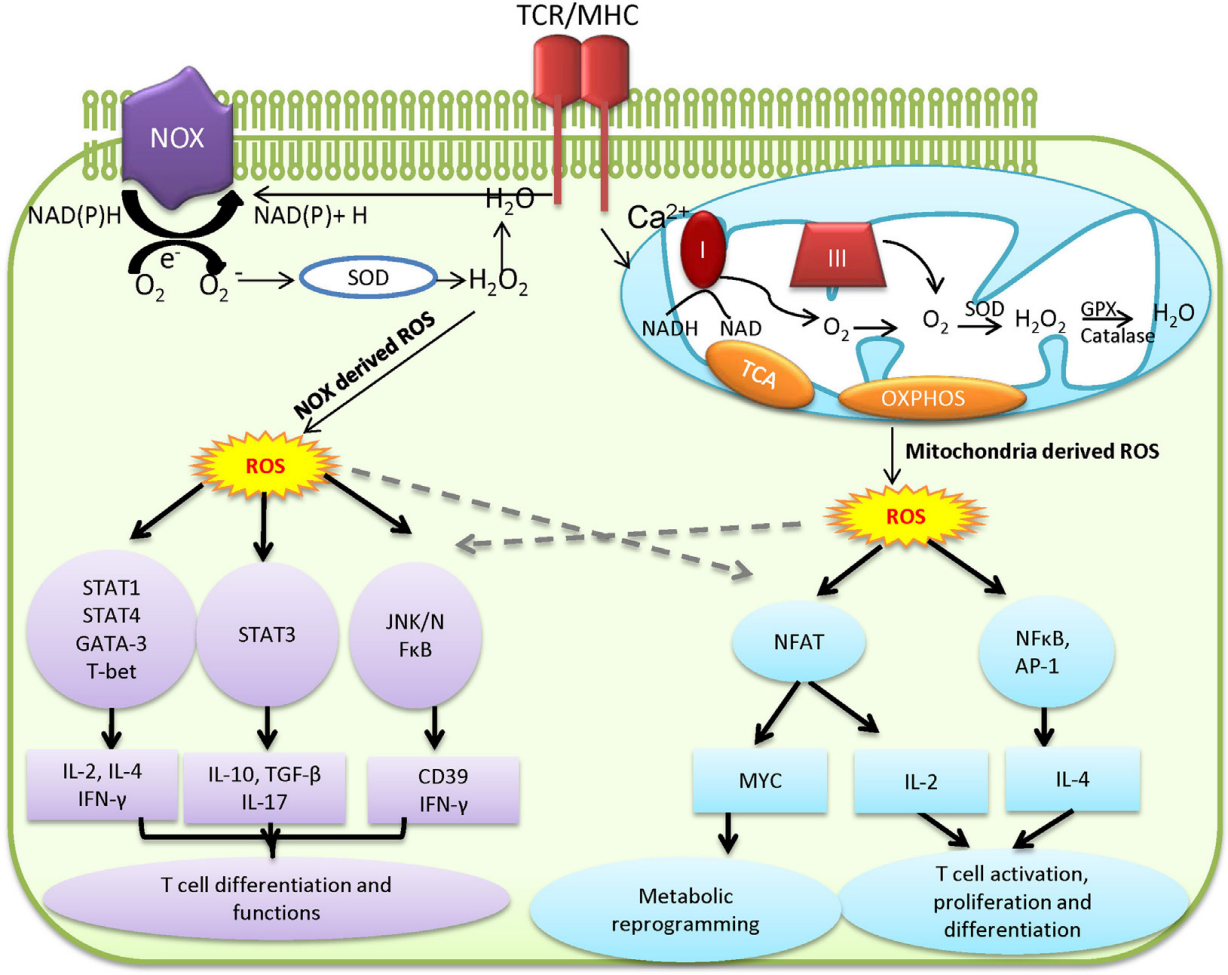
Mitochondria and NADPH oxidases (NOX)-derived reactive oxygen species (ROS) regulates T cell activation, differentiation, and metabolism. Mitochondria and NOX are the two major sources of ROS. The stimulation of T cell receptor (TCR) initiates signaling and metabolic events that drive ROS production in cytoplasm through NOX-dependent reaction and ROS production in mitochondria *via* mitochondria electron transport chain (ETC). Excess ROS causes damage and cell death. However, physiologically relevant levels of ROS mediate essential redox signaling through nodulation of a wide spectrum of redox-sensitive transcription factors to drive T cell activation and function.

T cell activation requires ligation of TCR and the major histocompatibility complex molecules. This interaction will initiate the signaling cascade and activation of transcriptional factors such as nuclear factor of activated T cells (NFAT), activator protein 1 (AP-1), and nuclear factor of kappa light chain enhancer in B cells (42). It has been reported that TCR ligation increases the production of ROS from OXPHOS and cytoplasmic ROS from NADPH oxidases (NOXs), a family of plasma membrane-associated oxidases (36, 40, 41). ROS-mediated signaling events are required for driving T cell activation, proliferation, and differentiation (Figure 1) (36, 41). T cells with reduced production of mitochondrial ROS display impaired production of interleukin-2 and antigen-specific proliferation, indicating an essential signaling role for mitochondrial ROS in driving optimal TCR signaling. The proximal TCR signaling machinery, including zeta chain-associated protein kinase 70, linker of activated T cell, SH2 domain-containing leukocyte protein, phospholipase Cγ1, and protein kinase Cθ, is involved in driving ROS production upon T cell activation (36, 41, 43). Conversely, physiologically relevant levels of ROS facilitate the activation of oxidation-dependent transcription factors, such as NF-κB and AP-1, which are required for driving essential signaling events to support T cell-mediated immune responses (44–46). However, excessive ROS production following ablation of *de novo* glutathione (GSH) synthesis suppresses the activity of mammalian target of rapamycin and the expression of transcription factors NFAT and c-MYC, the latter of which control metabolic reprogramming following T cell activation (15, 47, 48). Thus, T cells fail to meet their increased energy and biosynthetic needs and display compromised proliferation (48). In addition, uncontrolled ROS production is involved in the activation-induced T-cell death by affecting expression of apoptosis related genes including Bcl-2 and FasL and mitochondrial membrane potential (43, 49–52). NOX-derived ROS modulates the function of GATA-binding protein 3, signal transducer and activator of transcription, and T-box transcription factor to collectively control T cell activation and differentiation. T cells from NOX-deficient animals showed a skewed Th17 phenotype, whereas NOX-intact cells exhibited a preferred Th1 response (39, 53–55). In CD8 T cells, NOX-derived ROS is involved in regulating the production of IFN-γ and CD39 expression through c-Jun N-terminal kinase and NFκB signaling (40, 56). Importantly, the impact of ROS on T cell activation can be extended to the later T cell differentiation stages. Fine tuning of ROS is required for polarizing T cell in part by engaging lineage-specific transcription factors and modulating cytokine profiles, and consequently directs T cell-mediated inflammatory responses (39, 40, 53–55, 57–61).

## Metabolic Pathways in Modulating Antioxidant Capacities

**Figure 2:**
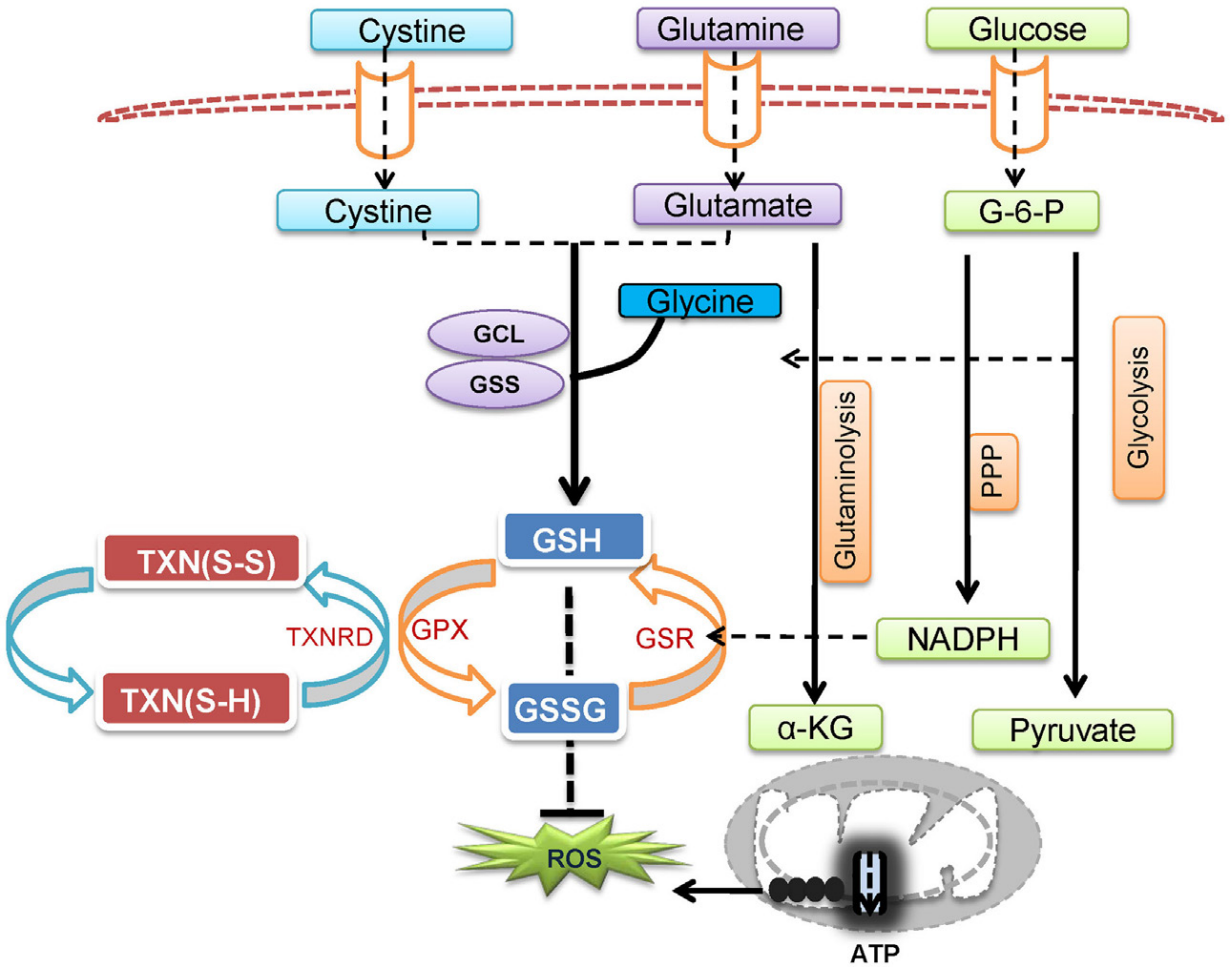
T cell metabolic programs that link to reactive oxygen species (ROS) production and the *de novo* synthesis of GSH. Pyruvate that is derived from glucose *via* glycolysis is shuttled to the mitochondria and drives the tricarboxylic acid (TCA) cycle and fuels oxidative phosphorylation (OXPHOS). Glucose-derived glucose-6-phosphate feeds into the pentose phosphate pathway (PPP) and produces NADPH in the cytoplasm. In addition, glutamate feeds the TCA cycle through α-ketoglutarate (α-KG) to fuel OXPHOS and generate ROS. Excessive ROS production is regulated by glutathione (GSH), a tripeptide of glutamine, cysteine, and glycine, which is synthesized *de novo* by glutamate-cysteine ligase (GCL) and glutathione synthetase (GSS). In addition, NADPH, glutathione-disulfide reductase (GSR), and glutathione peroxidase (GPX) are involved in regenerating GSH from glutathione disulfide (GSSG), whereas thioredoxin reductase (TXNRD) is responsible for the regeneration of thioredoxin (TXN) to control oxidative stress in T cell.

Excessive ROS production causes collateral damage to macromolecules, cellular organelles, and eventually necrosis, which can lead to uncontrolled hyper-inflammation and tissue damage. Thus, a fine-tuned balance between ROS production and antioxidant capacity ensures appropriate levels of intracellular ROS (Figure 2) (44, 55, 62). GSH, a tripeptide of glutamine, cysteine, and glycine, is the most abundant antioxidant capable of providing reducing equivalents and also serves as a versatile nucleophilic cofactor in a wide spectrum of metabolic reactions in aerobic organisms (63, 64). Thioredoxin (TXN) is a class of small redox proteins that are involved in modulating cell surface receptors and confers tolerance to oxidative stress in T cells (65–69). A reciprocal redox reaction can be coupled between these two antioxidant systems to act as a backup for each other under certain conditions (70–77). Supporting these findings, the inhibition of thioredoxin reductase (TXNRD) conferred an increased susceptibility of cancer cells to GSH depletion (78–80). Glutathione-disulfide reductase (GSR) regenerates GSH from its oxidized form, glutathione disulfide (GSSG), whereas TXNRD is responsible for the regeneration of TXN once it has been oxidized. Importantly, both GSR and TXNRD require NADPH as a reducing agent. Upon antigen stimulation, both PPP and glutaminolysis are significantly upregulated and further enhance T cell antioxidant capacities by generating NADPH through metabolic reactions that are controlled by glucose-6-phosphate dehydrogenase, phosphoglycerate dehydrogenase, malic enzyme 1, and isocitrate dehydrogenase 1. The intracellular GSH concentrations are normally in a range of three orders of magnitude higher than extracellular GSH. Even though some cells are able to recycle extracellular GSH, it may only play a minor role in maintaining intracellular GSH pool (63, 64, 81–86). By contrast, both the regeneration of GSH from GSSG (recycling pathway) and *de novo* synthesis of GSH, by glutamate-cysteine ligase (GCL) and glutathione synthase (GS), are required to maintain intracellular GSH levels (64, 87). The ligation of glutamate and cysteine to form dipeptide ϒ-glutamylcysteine (ϒ-GC) is the first and also the rate-limiting step of GSH *de novo* synthesis, which is controlled by ATP-dependent ligase GCL, a heterodimer of a catalytic subunit (GCLC) and modifier subunit (GCLM). Subsequently, GSH is formed by GS-mediated ligation of ϒ-GC and glycine (88, 89). Thus, the supply of intracellular cysteine, glycine, and glutamate must fulfill the need of *de novo* synthesis of GSH during T cell activation. Supporting this idea, the metabolic processes that are involved in providing three amino acids are tightly regulated upon T cell activation (13, 15, 90–92). Upon T cell activation, heightened glycolysis, PPP, and glutaminolysis intersect with the *de novo* synthesis of GSH through promoting cysteine uptake and providing glycine, glutamine, and NADPH (93–95). As such, the genetic abrogation of *de novo* synthesis of GSH, the glucose, or glutamine starvation significantly dampens T cell activation (10, 13, 15, 20, 48).

## Glutamine Catabolism in Coordinating the Production of ROS and GSH

Glutamine has been known as a key nutrient, which supports a diverse range of cellular functions (93–102). Glutamine provides high proportions of the energy from OXPHOS, provides precursors for various biosynthetic pathways, as a key nitrogen and carbon donor, and also is catabolized to various intermediate metabolites that have signaling roles in modulating cellular processes. In specialized cells, such as the cells of the nervous system, glutamine catabolism intersects with signaling networks to support the production of central neurotransmitters including glutamate, GABA, and aspartate (103–106). To meet bioenergetic and biosynthetic demand during T cell growth and proliferation, glutaminolysis replenishes the anapleurotic substrate α-KG that fuels OXPHOS *via* the TCA cycle and also provides sources of nitrogen and carbon to support the biosynthesis of nonessential amino acids, lipids, nucleotides, and polyamines (13, 15, 102, 107). Similar to cancer cells, *de novo* synthesis of GSH in T cells, which relies on glutamine to provide precursors, plays an essential role in suppressing oxidative stress. Accordingly, glutaminolysis is a branched pathway that consists of several paths, enabling energy production through oxidation and biomolecule production, including GSH through biosynthesis (93–95). While the ATP generating capacity of glutaminolysis is considered to be redundant with glucose oxidation and/or FAO, the oxidation of glutamine is indispensable for driving T cell proliferation and differentiation (13, 15, 102). However, enhanced glutamine oxidation in the mitochondria also increases the production of its by-product, mitochondrial ROS, the main source of cellular ROS in T cells (35, 37). Therefore, glutamate represents a key branch point in glutaminolysis that can be committed toward mitochondrial oxidation to produce ATP and ROS, or toward *de novo* synthesis of GSH to modulate redox balance and suppress oxidative stress. In addition, the high rate of glutaminolysis ensures that the capacity to supply glutamate, the most abundant intracellular metabolite in cells, exceeds the demand for glutamate from each of the downstream metabolic branches. The branched pathways in glutaminolysis enable the production of counteracting metabolites, i.e., ROS and GSH, from a common metabolic precursor, and permit a fine-tuned coordination between the metabolic flux shunted toward GSH synthesis and the metabolic flux shunted toward OXPHOS. Consistent with this idea, the overall high consumption rate of glutamine in proliferative cells is suggested to provide a sensitive and precise regulation on intermediate metabolites that can be committed toward several metabolic branches, hence permitting rapid responses to meet the demands for energy production or antioxidant production (99, 108). In addition to increasing antioxidant capacity, T cells may adapt by shifting glucose catabolism from OXPHOS toward aerobic glycolysis, which could provide biosynthetic precursors and rapidly produce ATP by the substrate level of phosphorylation.

## Conclusion and Perspective

**Figure 3:**
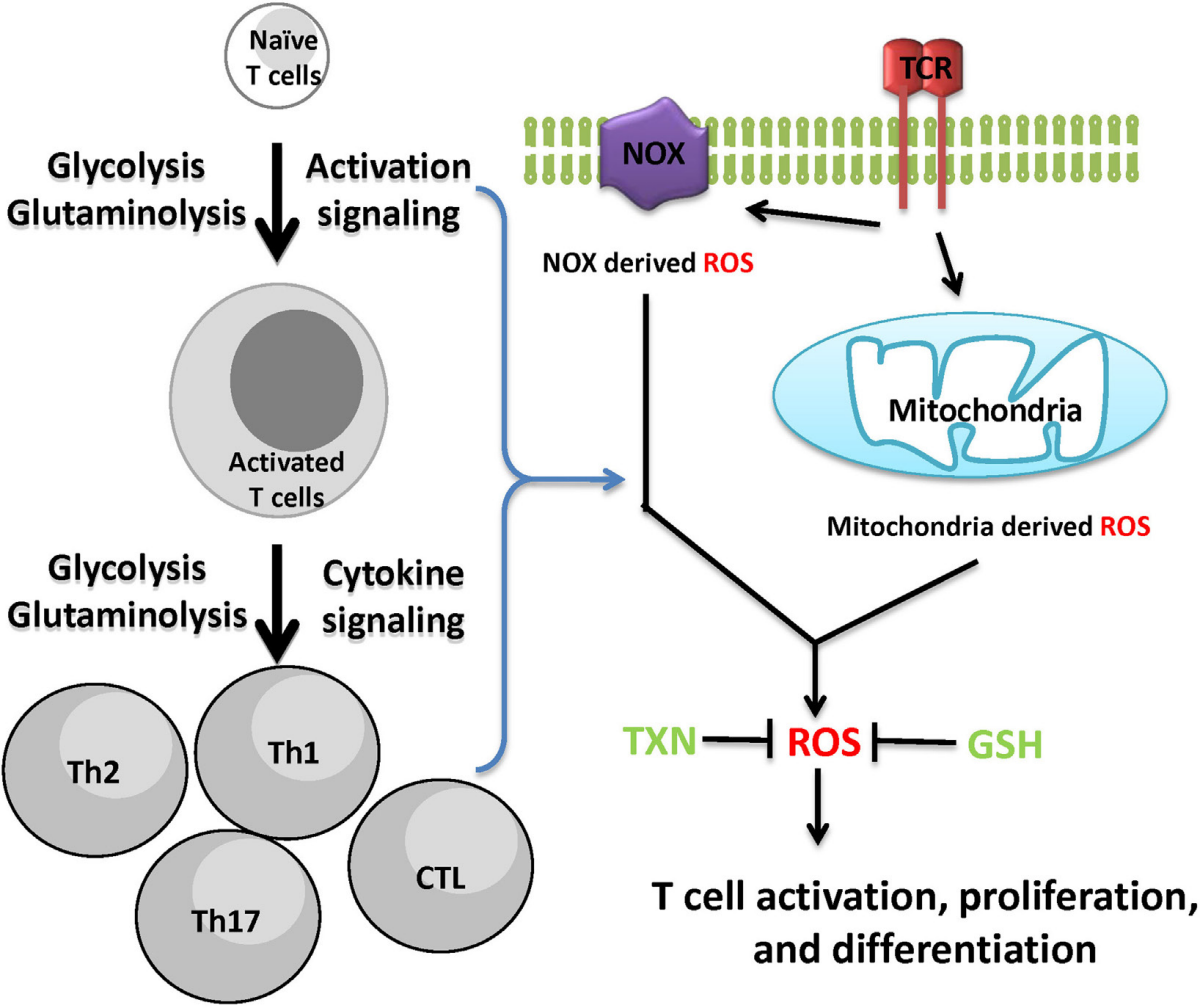
Cellular redox homeostasis is essential for mounting an effective T cell-mediated immune response. In addition to generate ATP and provide biosynthetic precursors, T cell activation-induced metabolic reprogramming actively regulates redox homeostasis. The coordination of *de novo* synthesis of glutathione (GSH) and the production of reactive oxygen species (ROS) ensures T cell redox balance and a fine-tuned T cell response.

Reactive oxygen species is not only a by-product of cellular metabolic programs but also a key signaling molecule involved in directing T cell activation and differentiation. However, uncontrolled ROS production causes collateral damage to biomolecules and cellular organelles. Under pathophysiological conditions, ROS generation from mitochondria can contribute to the initiation and progression of inflammatory and autoimmune diseases. However, oxidative stress caused by elevated ROS may also render key immune effector cells vulnerable to agents that can either modulate stress response or modulate metabolic pathways for ROS and GSH production (Figure 3). Redox signaling is essential to regulate T cell metabolism. Technological advancement in genetic models and metabolomics will allow us to understand the key metabolic processes that dictate T cell fate through modulation ROS and GSH production. Thus, further research is expected to illustrate the complex interplay between cellular metabolism and redox signaling in T cells, thereby offering novel therapies for treating inflammatory and autoimmune diseases.

## Author Contributions

JG and RHW wrote the manuscript. RW wrote and finalized the manuscript.

## Conflict of Interest Statement

The authors declare that the research was conducted in the absence of any commercial or financial relationships that could be construed as a potential conflict of interest.
